# Crystal structure of *S*-hexyl (*E*)-3-(4-methyl­benzyl­idene)di­thio­carbazate

**DOI:** 10.1107/S2056989015000080

**Published:** 2015-01-10

**Authors:** M. B. H. Howlader, M. S. Begum, M. C. Sheikh, R. Miyatake, E. Zangrando

**Affiliations:** a Department of Chemistry, Rajshahi University, Rajshahi-6205, Bangladesh; b Department of Applied Chemistry, Faculty of Engineering, University of Toyama, 3190 Gofuku, Toyama, 930-8555, Japan; c Center for Environmental Conservation and Research Safety, University of Toyama, 3190 Gofuku, Toyama, 930-8555, Japan; d Department of Chemical and Pharmaceutical Sciences, via Giorgieri 1, 34127, Trieste, Italy

**Keywords:** crystal structure, *S*-hexyl di­thio­carbazate, bidentate Schiff base, N—H⋯S hydrogen bonds, C—H⋯π inter­actions.

## Abstract

In the title compound, C_15_H_22_N_2_S_2_, the di­thio­carbazate group adopts an *E* conformation with respect to the C=N bond of the benzyl­idene moiety. In the crystal, mol­ecules are linked by pairs of N—H⋯S hydrogen bonds, forming inversion dimers with an *R* ^2^_2_(8) ring motif. The dimers are linked *via* C—H⋯π inter­actions, forming chains propagating along [100].

## Related literature   

For the biological properties of bidentate Schiff bases of *S*-methyl di­thio­carbazate or *S*-benzyl di­thio­carbaza­te and their bivalent metal complexes, see: Chan *et al.* (2008 ▸); How *et al.* (2008 ▸); Tarafder *et al.* (2002 ▸); Ali *et al.* (2002 ▸); Chew *et al.* (2004 ▸); Crouse *et al.* (2004 ▸). For their *N*,*S-*chelating behavior towards metal atoms, see for example: Islam *et al.* (2011 ▸). For the structures of related compounds, see: Tarafder *et al.* (2008 ▸, 2010 ▸).
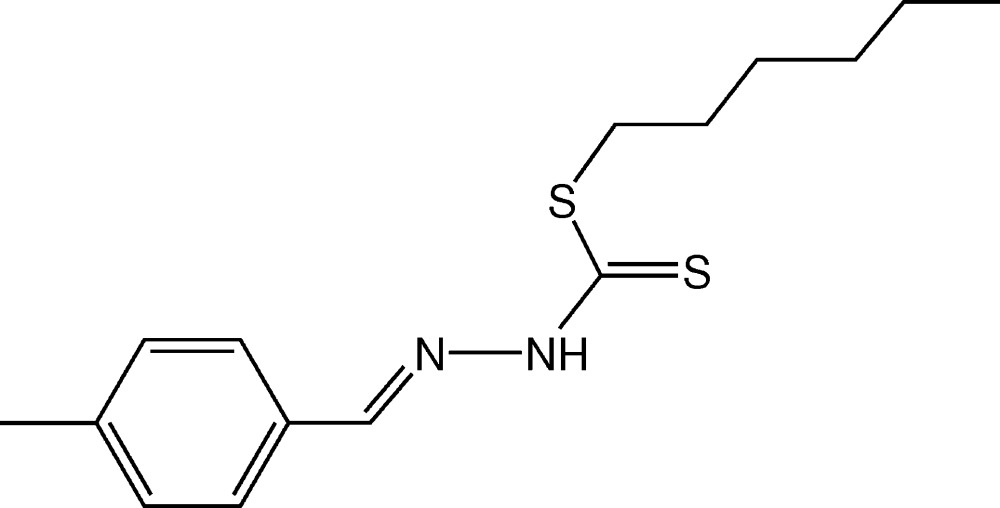


## Experimental   

### Crystal data  

C_15_H_22_N_2_S_2_
*M* *_r_* = 294.47
Triclinic, 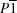

*a* = 4.79244 (9) Å
*b* = 11.3790 (2) Å
*c* = 14.5382 (3) Å
α = 100.1666 (7)°
β = 91.2117 (7)°
γ = 94.6754 (7)°
*V* = 777.26 (3) Å^3^
*Z* = 2
Cu *K*α radiation
μ = 3.00 mm^−1^
*T* = 173 K
0.19 × 0.11 × 0.07 mm

### Data collection  

Rigaku R-AXIS RAPID diffractometer
Absorption correction: multi-scan (*ABSCOR*; Rigaku, 2001 ▸) *T*_min_ = 0.615, *T*_max_ = 0.811
8970 measured reflections
2802 independent reflections
2162 reflections with *F* ^2^ > 2.0σ(*F* ^2^)
*R*_int_ = 0.052

### Refinement  

*R*[*F* ^2^ > 2σ(*F* ^2^)] = 0.046
*wR*(*F* ^2^) = 0.129
*S* = 1.04
2802 reflections
176 parameters
H atoms treated by a mixture of independent and constrained refinement
Δρ_max_ = 0.43 e Å^−3^
Δρ_min_ = −0.26 e Å^−3^

### 

Data collection: *RAPID-AUTO* (Rigaku, 2001 ▸); cell refinement: *RAPID-AUTO*; data reduction: *RAPID-AUTO*; program(s) used to solve structure: *SIR92* (Altomare *et al.*, 1994 ▸); program(s) used to refine structure: *SHELXL97* (Sheldrick, 2008 ▸); molecular graphics: *CrystalStructure* (Rigaku, 2010 ▸); software used to prepare material for publication: *CrystalStructure*.

## Supplementary Material

Crystal structure: contains datablock(s) General, I. DOI: 10.1107/S2056989015000080/su5050sup1.cif

Structure factors: contains datablock(s) I. DOI: 10.1107/S2056989015000080/su5050Isup2.hkl

Supporting information file. DOI: 10.1107/S2056989015000080/su5050Isup3.cml

**Fig. 1.**
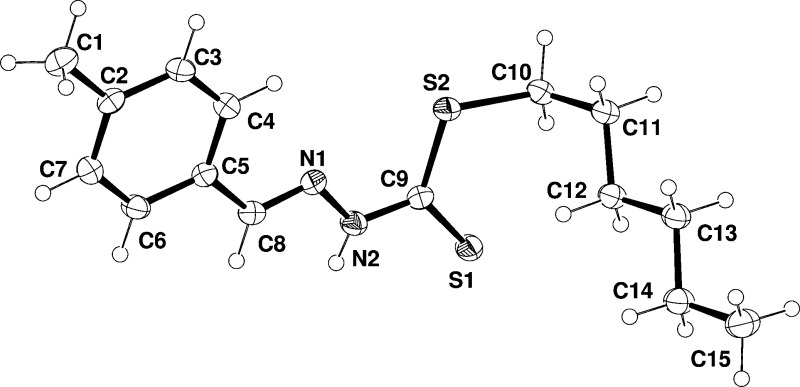
A view of the molecular structure of the title compound, with atom labelling. Displacement ellipsoids are drawn at the 50% probability level.

**Fig. 2.**
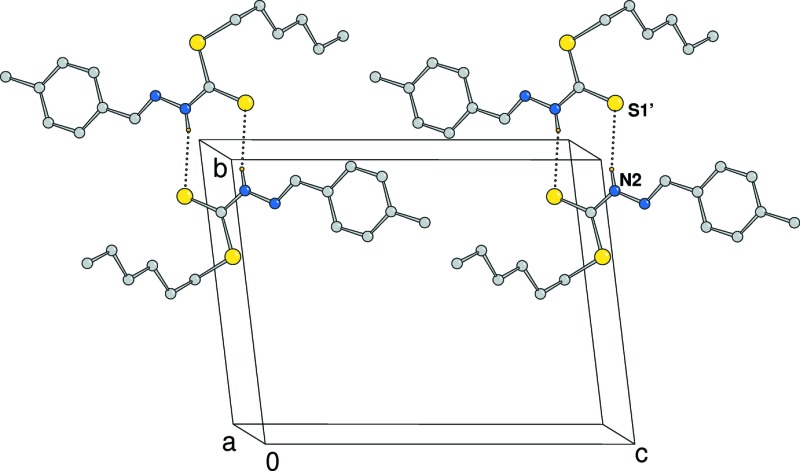
A partial view along the a axis of the crystal packing of the title compound. The hydrogen bonds are shown as dashed lines (see Table 1 for details; H atoms not involved in hydrogen bonding have been omitted for clarity).

. DOI: 10.1107/S2056989015000080/su5050fig1.tif
A view of the mol­ecular structure of the title compound, with atom labelling. Displacement ellipsoids are drawn at the 50% probability level.

a . DOI: 10.1107/S2056989015000080/su5050fig2.tif
A partial view along the *a* axis of the crystal packing of the title compound. The hydrogen bonds are shown as dashed lines (see Table 1 for details; H atoms not involved in hydrogen bonding have been omitted for clarity).

CCDC reference: 1035819

Additional supporting information:  crystallographic information; 3D view; checkCIF report

## Figures and Tables

**Table 1:** Hydrogen-bond geometry (, ) *Cg*1 is the centroid of the C1C6 ring.

*D*H*A*	*D*H	H*A*	*D* *A*	*D*H*A*
N2H9S1^i^	0.83(3)	2.56(3)	3.3760(19)	168(2)
C1H2*Cg*1^ii^	0.98	2.61	3.529(3)	157

Symmetry codes: (i) 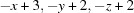; (ii) 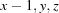.

## supplementary crystallographic information

### S1. Synthesis and crystallization

To an ethano­lic solution of KOH (2.81 g, 0.05 mol) hydrazine hydrate (2.50 g, 0.05 mol, 99%) was added and the mixture was stirred at 273 K. To this solution carbon di­sulfide (3.81 g, 0.05 mol) was added drop wise with constant stirring for one hour. Then *n*-bromo­hexane (8.25 g, 0.05 mol) was added drop wise with vigorous stirring at 273 K for an additional hour. Finally, 4-methyl­benzaldehyde (6.0 g, 0.05 mol) in ethanol was added and the mixture refluxed for 30 min. The mixture was filtered while hot and then the filtrate was cooled to 273 K giving a precipitate of the Schiff base product. It was recrystallized from ethanol at room temperature and dried in a vacuum desiccator over anhydrous CaCl_2_. Colourless crystals, suitable for X-ray diffraction, of the title compound were obtained by slow evaporation of a solution in ethanol/aceto­nitrile (2:1) after 23 days (m.p.: 357 K).

### S2. Refinement

Crystal data, data collection and structure refinement details are summarized in Table 2. The H atom of the NH group was located in a difference Fourier map and freely refined. The C-bound H atoms were included in calculated positions and treated as riding atoms: C—H = 0.95–0.99 Å with *U*_iso_(H) = 1.5*U*_eq_(C) for methy H atoms and = 1.2*U*_eq_(C) for other H atoms.

### S3. Comment

Bidentate Schiff bases of S-methyl dithiocarbazate or S-benzyl dithiocarbazates and their bivalent metal complexes have received considerable attention in the field of medical science for their antibacterial, antifungal, antiviral, antitumour, and anticancer activities (Chan *et al.*, 2008; How *et al.*, 2008; Tarafder *et al.*, 2002; Ali *et al.*, 2002; Chew *et al.*, 2004; Crouse *et al.*, 2004)

The molecular structure of the title compound is shown in Fig. 1. The Schiff base exists in the thione tautomeric form with the dithiocarbazate fragment adopting an *E* conformation with respect to the C═N bond of the benzylidene moiety. The *β*-nitrogen and the thioketo sulphur are *trans* located with respect to the C9—N2 bond. The bond lengths and angles are within the normal ranges and are comparable to those in related structures (Tarafder *et al.*, 2008, 2010). The molecule is in its thione tautomeric and the co-planarity of atoms (with the exception of the S-hexyl chain) indicates an electron delocalization within it. The molecule, when used in coordination chemistry, requires a rotation about the C9—N2 by 180 ° in order to allow the N,S chelating behavior towards the metal atom (Islam *et al.*, 2011).

In the crystal, molecules are linked by pairs of N—H···S hydrogen bonds forming inversion dimers with an R^2^_2_(8) ring motif (Table 1 and Fig. 2). The dimers are linked via C—H···π interactions forming chains propagating along the *a* axis direction (Table 1).

### Crystal data

**Table:**

C_15_H_22_N_2_S_2_	*Z* = 2
*M**_r_* = 294.47	*F*(000) = 316.00
Triclinic, *P*1	*D*_x_ = 1.258 Mg m^−^^3^
Hall symbol: -P 1	Cu *K*α radiation, λ = 1.54187 Å
*a* = 4.79244 (9) Å	Cell parameters from 7029 reflections
*b* = 11.3790 (2) Å	θ = 3.1–68.2°
*c* = 14.5382 (3) Å	µ = 3.00 mm^−^^1^
α = 100.1666 (7)°	*T* = 173 K
β = 91.2117 (7)°	Prism, colorless
γ = 94.6754 (7)°	0.19 × 0.11 × 0.07 mm
*V* = 777.26 (3) Å^3^	

### Data collection

**Table:**

Rigaku R-AXIS RAPID diffractometer	2162 reflections with *F*^2^ > 2.0σ(*F*^2^)
Detector resolution: 10.000 pixels mm^-1^	*R*_int_ = 0.052
ω scans	θ_max_ = 68.2°
Absorption correction: multi-scan (*ABSCOR*; Rigaku, 2001)	*h* = −5→5
*T*_min_ = 0.615, *T*_max_ = 0.811	*k* = −13→13
8970 measured reflections	*l* = −17→17
2802 independent reflections	

### Refinement

**Table:**

Refinement on *F*^2^	Secondary atom site location: difference Fourier map
*R*[*F*^2^ > 2σ(*F*^2^)] = 0.046	Hydrogen site location: inferred from neighbouring sites
*wR*(*F*^2^) = 0.129	H atoms treated by a mixture of independent and constrained refinement
*S* = 1.04	*w* = 1/[σ^2^(*F*_o_^2^) + (0.0784*P*)^2^] where *P* = (*F*_o_^2^ + 2*F*_c_^2^)/3
2802 reflections	(Δ/σ)_max_ < 0.001
176 parameters	Δρ_max_ = 0.43 e Å^−^^3^
0 restraints	Δρ_min_ = −0.26 e Å^−^^3^
Primary atom site location: structure-invariant direct methods	

### Special details

**Table:**

Experimental. All e.s.d.'s (except the e.s.d. in the dihedral angle between two l.s. planes) are estimated using the full covariance matrix. The cell e.s.d.'s are taken into account individually in the estimation of e.s.d.'s in distances, angles and torsion angles; correlations between e.s.d.'s in cell parameters are only used when they are defined by crystal symmetry. An approximate (isotropic) treatment of cell e.s.d.'s is used for estimating e.s.d.'s involving l.s. planes.
Geometry. ENTER SPECIAL DETAILS OF THE MOLECULAR GEOMETRY
Refinement. Refinement was performed using all reflections. The weighted *R*-factor (*wR*) and goodness of fit (*S*) are based on *F*^2^. *R*-factor (gt) are based on *F*. The threshold expression of *F*^2^ > 2.0 σ(*F*^2^) is used only for calculating *R*-factor (gt).

### Fractional atomic coordinates and isotropic or equivalent isotropic displacement parameters (Å^2^)

**Table:**

	*x*	*y*	*z*	*U*_iso_*/*U*_eq_	
S1	1.54116 (12)	0.83289 (5)	0.90597 (4)	0.0326 (2)	
S2	1.17201 (12)	0.64667 (5)	0.98677 (4)	0.0333 (2)	
N1	1.0048 (4)	0.84542 (15)	1.10436 (12)	0.0293 (5)	
N2	1.2012 (4)	0.87708 (16)	1.04349 (13)	0.0297 (5)	
C1	0.0848 (5)	0.8428 (3)	1.42942 (17)	0.0390 (6)	
C2	0.2994 (5)	0.8663 (2)	1.35925 (15)	0.0300 (5)	
C3	0.3922 (5)	0.77235 (19)	1.29544 (15)	0.0333 (6)	
C4	0.5864 (5)	0.79268 (19)	1.23002 (15)	0.0327 (6)	
C5	0.6987 (5)	0.90853 (18)	1.22664 (14)	0.0282 (5)	
C6	0.6060 (5)	1.00311 (19)	1.28972 (15)	0.0314 (6)	
C7	0.4103 (5)	0.9817 (2)	1.35483 (15)	0.0334 (6)	
C8	0.9098 (5)	0.93191 (19)	1.15966 (15)	0.0281 (5)	
C9	1.3050 (5)	0.79357 (19)	0.98029 (15)	0.0290 (5)	
C10	1.3277 (5)	0.55512 (19)	0.88956 (15)	0.0329 (6)	
C11	1.1314 (5)	0.51925 (18)	0.80356 (15)	0.0333 (6)	
C12	1.0591 (5)	0.62287 (19)	0.75707 (15)	0.0329 (6)	
C13	0.8582 (5)	0.58746 (19)	0.67205 (15)	0.0330 (6)	
C14	0.7870 (5)	0.69523 (19)	0.63000 (16)	0.0363 (6)	
C15	0.5920 (6)	0.6635 (3)	0.54445 (17)	0.0445 (7)	
H1	0.0707	0.7576	1.4338	0.0468*	
H2	−0.0975	0.8647	1.4095	0.0468*	
H3	0.1417	0.8908	1.4907	0.0468*	
H4	0.3201	0.6925	1.2971	0.0400*	
H5	0.6440	0.7270	1.1869	0.0393*	
H6	0.6778	1.0830	1.2880	0.0377*	
H7	0.3505	1.0474	1.3974	0.0401*	
H8	0.9772	1.0121	1.1572	0.0337*	
H9	1.266 (5)	0.948 (3)	1.0471 (17)	0.044 (8)*	
H10	1.4987	0.6000	0.8723	0.0395*	
H11	1.3848	0.4817	0.9098	0.0395*	
H12	1.2193	0.4603	0.7573	0.0400*	
H13	0.9556	0.4791	0.8220	0.0400*	
H14	1.2346	0.6621	0.7376	0.0394*	
H15	0.9745	0.6826	0.8037	0.0394*	
H16	0.9442	0.5300	0.6240	0.0396*	
H17	0.6834	0.5467	0.6907	0.0396*	
H18	0.6990	0.7519	0.6781	0.0435*	
H19	0.9628	0.7367	0.6129	0.0435*	
H20	0.5574	0.7366	0.5206	0.0534*	
H21	0.4142	0.6253	0.5612	0.0534*	
H22	0.6782	0.6081	0.4961	0.0534*	

### Atomic displacement parameters (Å^2^)

**Table:**

	*U*^11^	*U*^22^	*U*^33^	*U*^12^	*U*^13^	*U*^23^
S1	0.0382 (4)	0.0272 (4)	0.0329 (4)	0.0014 (3)	0.0096 (3)	0.0062 (3)
S2	0.0453 (5)	0.0245 (3)	0.0317 (4)	0.0021 (3)	0.0077 (3)	0.0087 (3)
N1	0.0353 (12)	0.0273 (10)	0.0259 (10)	0.0011 (9)	0.0034 (9)	0.0070 (8)
N2	0.0342 (12)	0.0236 (10)	0.0318 (11)	0.0010 (9)	0.0072 (9)	0.0062 (8)
C1	0.0374 (15)	0.0452 (14)	0.0383 (14)	0.0048 (12)	0.0056 (11)	0.0169 (11)
C2	0.0308 (14)	0.0345 (12)	0.0266 (12)	0.0037 (10)	−0.0019 (10)	0.0104 (10)
C3	0.0404 (15)	0.0240 (11)	0.0361 (13)	−0.0023 (10)	0.0017 (11)	0.0091 (10)
C4	0.0413 (15)	0.0255 (11)	0.0312 (12)	0.0045 (11)	0.0007 (11)	0.0039 (10)
C5	0.0324 (14)	0.0268 (11)	0.0263 (12)	0.0034 (10)	−0.0014 (10)	0.0071 (9)
C6	0.0350 (14)	0.0238 (11)	0.0364 (13)	0.0016 (10)	0.0032 (11)	0.0079 (10)
C7	0.0399 (15)	0.0290 (12)	0.0314 (12)	0.0060 (11)	0.0044 (11)	0.0033 (10)
C8	0.0296 (13)	0.0255 (11)	0.0304 (12)	0.0025 (10)	0.0003 (10)	0.0083 (9)
C9	0.0333 (14)	0.0280 (11)	0.0274 (11)	0.0054 (10)	−0.0011 (10)	0.0084 (9)
C10	0.0406 (15)	0.0249 (11)	0.0346 (13)	0.0086 (11)	0.0042 (11)	0.0061 (10)
C11	0.0416 (15)	0.0245 (11)	0.0350 (13)	0.0034 (11)	0.0046 (11)	0.0074 (10)
C12	0.0396 (15)	0.0254 (11)	0.0345 (13)	0.0044 (10)	0.0050 (11)	0.0069 (10)
C13	0.0362 (14)	0.0272 (12)	0.0364 (13)	0.0021 (10)	0.0056 (11)	0.0077 (10)
C14	0.0416 (16)	0.0301 (12)	0.0388 (13)	0.0035 (11)	0.0048 (12)	0.0100 (10)
C15	0.0494 (17)	0.0415 (14)	0.0449 (15)	0.0004 (13)	−0.0021 (13)	0.0160 (12)

### Geometric parameters (Å, º)

**Table:**

S1—C9	1.670 (3)	C1—H2	0.980
S2—C9	1.759 (3)	C1—H3	0.980
S2—C10	1.814 (3)	C3—H4	0.950
N1—N2	1.375 (3)	C4—H5	0.950
N1—C8	1.277 (3)	C6—H6	0.950
N2—C9	1.335 (3)	C7—H7	0.950
C1—C2	1.505 (4)	C8—H8	0.950
C2—C3	1.395 (3)	C10—H10	0.990
C2—C7	1.389 (4)	C10—H11	0.990
C3—C4	1.380 (4)	C11—H12	0.990
C4—C5	1.392 (3)	C11—H13	0.990
C5—C6	1.394 (3)	C12—H14	0.990
C5—C8	1.460 (4)	C12—H15	0.990
C6—C7	1.384 (4)	C13—H16	0.990
C10—C11	1.524 (3)	C13—H17	0.990
C11—C12	1.518 (4)	C14—H18	0.990
C12—C13	1.526 (3)	C14—H19	0.990
C13—C14	1.523 (4)	C15—H20	0.980
C14—C15	1.512 (4)	C15—H21	0.980
N2—H9	0.84 (3)	C15—H22	0.980
C1—H1	0.980		
			
C9—S2—C10	103.78 (11)	C2—C7—H7	119.297
N2—N1—C8	115.97 (18)	C6—C7—H7	119.294
N1—N2—C9	120.61 (18)	N1—C8—H8	119.724
C1—C2—C3	120.9 (2)	C5—C8—H8	119.729
C1—C2—C7	121.6 (2)	S2—C10—H10	108.909
C3—C2—C7	117.5 (2)	S2—C10—H11	108.910
C2—C3—C4	121.5 (2)	C11—C10—H10	108.900
C3—C4—C5	120.6 (2)	C11—C10—H11	108.913
C4—C5—C6	118.3 (2)	H10—C10—H11	107.737
C4—C5—C8	121.55 (19)	C10—C11—H12	108.647
C6—C5—C8	120.14 (19)	C10—C11—H13	108.645
C5—C6—C7	120.6 (2)	C12—C11—H12	108.656
C2—C7—C6	121.4 (2)	C12—C11—H13	108.654
N1—C8—C5	120.5 (2)	H12—C11—H13	107.587
S1—C9—S2	126.25 (13)	C11—C12—H14	108.631
S1—C9—N2	120.30 (17)	C11—C12—H15	108.636
S2—C9—N2	113.44 (17)	C13—C12—H14	108.637
S2—C10—C11	113.33 (17)	C13—C12—H15	108.627
C10—C11—C12	114.44 (17)	H14—C12—H15	107.578
C11—C12—C13	114.52 (18)	C12—C13—H16	109.166
C12—C13—C14	112.20 (17)	C12—C13—H17	109.171
C13—C14—C15	113.80 (18)	C14—C13—H16	109.159
N1—N2—H9	121.2 (17)	C14—C13—H17	109.167
C9—N2—H9	118.1 (17)	H16—C13—H17	107.879
C2—C1—H1	109.474	C13—C14—H18	108.795
C2—C1—H2	109.467	C13—C14—H19	108.797
C2—C1—H3	109.465	C15—C14—H18	108.803
H1—C1—H2	109.474	C15—C14—H19	108.808
H1—C1—H3	109.480	H18—C14—H19	107.672
H2—C1—H3	109.467	C14—C15—H20	109.472
C2—C3—H4	119.227	C14—C15—H21	109.469
C4—C3—H4	119.232	C14—C15—H22	109.470
C3—C4—H5	119.709	H20—C15—H21	109.471
C5—C4—H5	119.710	H20—C15—H22	109.473
C5—C6—H6	119.686	H21—C15—H22	109.472
C7—C6—H6	119.692		
			
C9—S2—C10—C11	−99.27 (15)	C3—C4—C5—C6	−1.2 (4)
C10—S2—C9—S1	−4.68 (19)	C3—C4—C5—C8	178.37 (19)
C10—S2—C9—N2	176.19 (15)	C4—C5—C6—C7	0.9 (4)
N2—N1—C8—C5	179.48 (17)	C4—C5—C8—N1	−2.6 (4)
C8—N1—N2—C9	−177.29 (18)	C6—C5—C8—N1	176.96 (19)
N1—N2—C9—S1	179.41 (16)	C8—C5—C6—C7	−178.62 (18)
N1—N2—C9—S2	−1.4 (3)	C5—C6—C7—C2	−0.3 (4)
C1—C2—C3—C4	179.25 (19)	S2—C10—C11—C12	66.6 (2)
C1—C2—C7—C6	−179.50 (19)	C10—C11—C12—C13	−178.90 (17)
C3—C2—C7—C6	−0.1 (4)	C11—C12—C13—C14	178.41 (17)
C7—C2—C3—C4	−0.2 (4)	C12—C13—C14—C15	178.99 (17)
C2—C3—C4—C5	0.8 (4)		

### Hydrogen-bond geometry (Å, º)

Cg1 is the centroid of the C1–C6 ring.

**Table:**

*D*—H···*A*	*D*—H	H···*A*	*D*···*A*	*D*—H···*A*
N2—H9···S1^i^	0.83 (3)	2.56 (3)	3.3760 (19)	168 (2)
C1—H2···*Cg*1^ii^	0.98	2.61	3.529 (3)	157

Symmetry codes: (i) −*x*+3, −*y*+2, −*z*+2; (ii) *x*−1, *y*, *z*.
