# Supplementary figures and images for: Crystal structure of S-hexyl (E)-3-(4-methyl­benzyl­idene)di­thio­carbazate
Source: Acta Crystallogr E Crystallogr Commun. 2015 Jan 10;71(Pt 2):o103–4. doi: 10.1107/S2056989015000080 (PMC4384598; doi:10.1107/S2056989015000080)

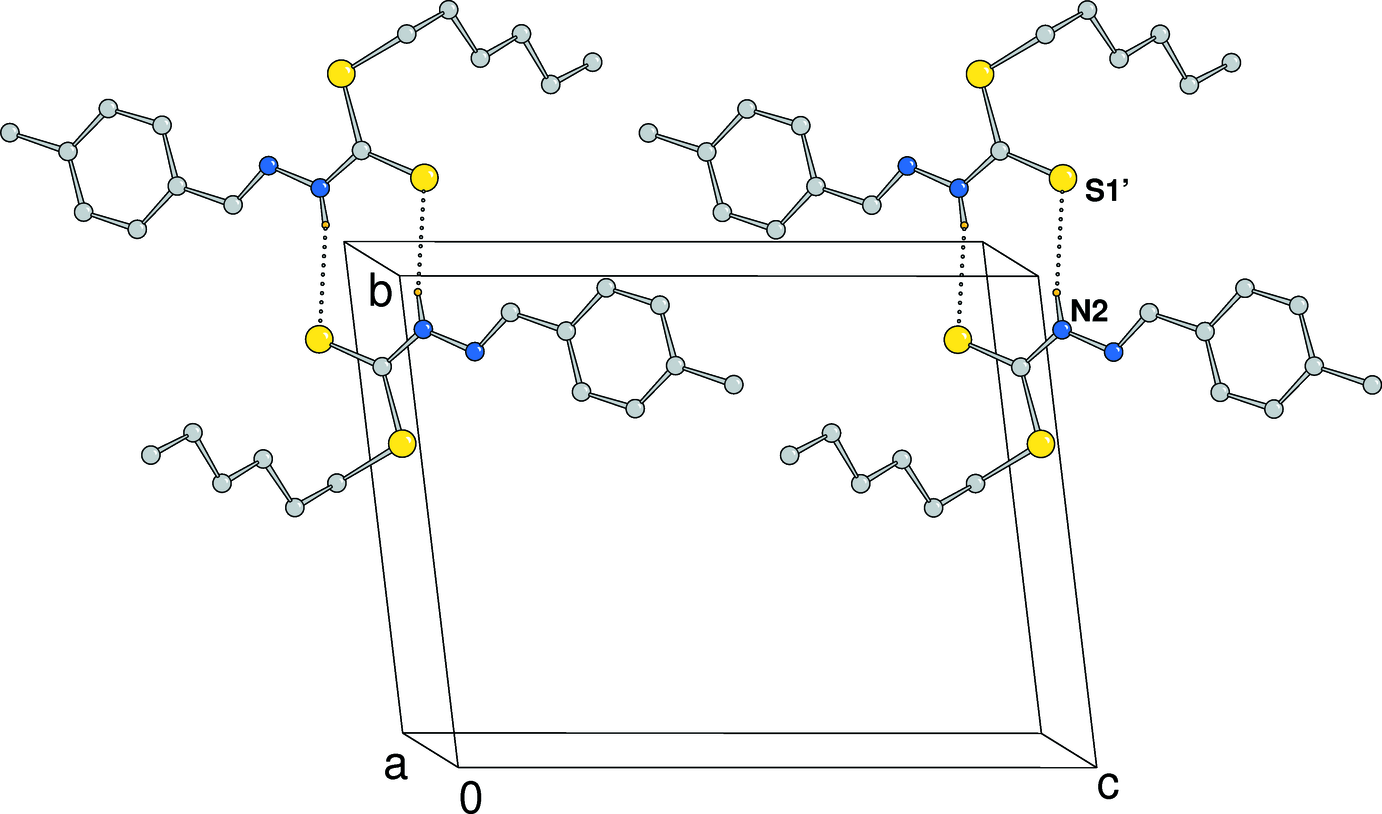

Supplement: Supplementary file 5 [file e-71-0o103-fig2.tif]
